# Generation of heterozygous and homozygous NF1 lines from human-induced pluripotent stem cells using CRISPR/Cas9 to investigate bone defects associated with neurofibromatosis type 1

**DOI:** 10.3389/fcell.2024.1359561

**Published:** 2024-02-28

**Authors:** Annabelle Darle, Thibault Mahiet, Déborah Aubin, Manon Doyen, Lina El Kassar, Béatrice Parfait, Gilles Lemaitre, Christine Baldeschi, Jennifer Allouche, Nathalie Holic

**Affiliations:** ^1^ Centre d’Etude des Cellules Souches, Corbeil-Essonnes, France; ^2^ Université Paris-Saclay, Université d’Evry, Corbeil-Essonnes, France; ^3^ INSERM U861, I-Stem, Association Française contre les Myopathies (AFM), Institute for Stem Cell Therapy and Exploration of Monogenic Diseases, Corbeil-Essonnes, France; ^4^ Phenocell SAS, Grasse, France; ^5^ Equipe “Génomique et Epigénétique des Tumeurs Rares”, UMR INSERM 1016 & Université Paris Cité, Institut Cochin, Paris, France; ^6^ GHU AP-HP Centre-Université Paris Cité, Fédération de Médecine Génomique, Service de Médecine Génomique des Maladies de Système et d'Organe, Hôpital Cochin, Paris, France

**Keywords:** neurofibromatosis type I, gene editing, CRISPR/Cas9, human-induced pluripotent stem cells, osteogenic differentiation, disease modeling

## Abstract

Neurofibromatosis type 1 (NF1) is one of the most common genetic disorders caused by heterozygous germline *NF1* mutations. NF1 affects many systems, including the skeletal system. To date, no curative therapies are available for skeletal manifestations such as scoliosis and tibial dysplasia, mainly due to the lack of knowledge about the mechanisms that underlie this process. By using CRISPR/Cas9-mediated gene editing in human-induced pluripotent stem cells (hiPSCs) to minimize the variability due to genetic background and epigenetic factors, we generated isogenic heterozygous and homozygous *NF1*-deficient hiPSC lines to investigate the consequences of neurofibromin inactivation on osteoblastic differentiation. Here, we demonstrate that loss of one or both copies of *NF1* does not alter the potential of isogenic hiPSCs to differentiate into mesenchymal stem cells (hiPSC-MSCs). However, *NF1* (+/−) and *NF1* (−/−) hiPSC-MSCs show a defect in osteogenic differentiation and mineralization. In addition, we show that a mono-allelic deletion in *NF1* in an isogenic context is sufficient to impair cell differentiation into osteoblasts. Overall**,** this study highlights the relevance of generating isogenic lines, which may help in genotype–phenotype correlation and provide a human cellular model to understand the molecular mechanisms underlying NF1 and, thus, discover new therapeutic strategies.

## 1 Introduction

Neurofibromatosis type 1 (NF1) is the most common genetic disorder caused by *NF1* gene mutations that encode neurofibromin, which is ubiquitously expressed. NF1 involves multiple systems including the skin, eyes, brain, and skeletal system (Legius et al., 2021). A total of 20%–50% of NF1 patients present skeletal abnormalities that impact their life quality (Elefteriou et al., 2009; Ma et al., 2020). These include osteopenia, osteoporosis, short stature, dystrophic scoliosis, and tibia bowing that lead to fracture and tibial pseudarthrosis. There are currently no therapeutic options for skeletal manifestations. The management of complications such as pseudarthrosis and scoliosis includes surgical interventions (Elefteriou et al., 2009; Bergqvist et al., 2020). It is therefore necessary to understand the molecular mechanisms associated with pathological defects in order to develop new therapeutic options. The mechanisms by which *NF1* mutations promote bone fragility are not well understood. The impact of *NF1* inactivation on skeletal manifestations has mainly been studied in genetically engineered NF1 mouse models. Mice carrying a mono-allelic mutation in the *NF1* gene do not develop osseous phenotypes (Jacks et al., 1994). The inactivation of both *NF1* alleles in all cell types of mice leads to embryonic lethality due to heart and neural crest derivative defects (Brannan et al., 1994; Yzaguirre et al., 2015). Conditional mouse models lacking both *NF1* alleles in bone cells or in mesenchymal progenitor cells show skeletal defects that include decreased bone mass, tibial bowling, and delayed consolidation of fractures (Elefteriou et al., 2006; Kolanczyk et al., 2007; Wang et al., 2011; Zhang et al., 2011; Paria et al., 2023). All these mouse models have provided important and valuable insights into the complex biology of NF1. To complement NF1 mouse models, studies based on human primary NF1 mesenchymal stem cells (MSCs) isolated from NF1 patient biopsies with skeletal abnormalities showed that the loss of neurofibromin impairs the differentiation of MSCs into osteoblasts (Leskelä et al., 2009; Sharma et al., 2013; Li et al., 2019). Nevertheless, obtaining bone biopsies is technically challenging and is highly invasive for patients.

Human-induced pluripotent stem cells (hiPSCs) represent an interesting alternative to primary cells. Because of their property of self-renewal and their pluripotency, hiPSCs have been widely used for disease modeling over the past decade (Liu et al., 2018; Karagiannis et al., 2019). NF1 patient-derived hiPSCs have already been generated to study clinical manifestations associated with NF1 patients, including plexiform neurofibromas or other NF1-associated brain and nerve pathologies (Wegscheid et al., 2018; Carrió et al., 2019; Anastasaki et al., 2020; Mo et al., 2021). However, no study using *NF1* hiPSCs has been conducted on the origin and pathological mechanisms of bone defects. More recently, gene editing technology, including CRISPR/Cas9 (clustered regularly interspaced short palindromic repeat/CRISPR-associated protein 9) has provided a powerful tool for modeling various human genetic diseases by engineering human isogenic hiPSC lines that share a genetic background similar to that of parental cells, except for the mutation (Wong et al., 2023). As bone defects have previously been suggested to result from the localized bi-allelic inactivation of *NF1* due to somatic loss of heterozygosity (Stevenson et al., 2006; Lee et al., 2012), we generated *NF1* (+/−) and *NF1* (−/−) isogenic hiPSC lines using CRISPR/Cas9 technology. Human isogenic iPSC lines were then differentiated into mesenchymal stem cells (MSCs) to assess the impact of *NF1* loss on osteoblast differentiation. Altogether, our data suggest that loss of a single allele of *NF1* in an isogenic context is sufficient to impair osteoblast differentiation, as shown by the reduction of osteoblastic markers at the mRNA level and the reduction of alkaline phosphatase activity. All these results were confirmed in hiPSCs derived from NF1 patients. The *NF1* (−/−) isogenic line generated by CRISPR-Cas9 opens perspectives that can clarify the consequences of the bi-allelic *NF1* gene inactivation in bone defects, and therefore improve the development of therapeutics for NF1 patients.

## 2 Materials and methods

### 2.1 Pluripotent stem cell reprogramming and culture

*NF1*-1 and *NF1*-2 hiPSC lines were reprogrammed using OSKM episomal technique by Phenocell from two patient-derived fibroblasts carrying *NF1* mutations. Approval was received from the local committee of the Ile de France (Declaration DC-2010-1101/CPP dossier n° Am6136-2-COL2828), and written informed consent was received from patients. *NF1*-1 and *NF1*-2 hiPSC lines carry the nonsense heterozygous mutation c.1721 + 3A>G located at intron 15 in the *NF1* gene and c.2412del at exon 21, respectively, leading to the formation of a premature stop codon resulting in the expression of a truncated neurofibromin. Two control *NF1* (+/+) hiPSC lines (*WT*-1 and *WT*-2) purchased from Phenocell were also generated from control donors. The parental (+/+) control hiPSC line used for Crispr/Cas9 engineering corresponds to the *WT*-1 line. HiPSCs were maintained and expanded with the StemMACS™ iPS-Brew XF medium (Miltenyi Biotec) on Matrigel^®^ hESC-qualified Matrix (Corning)-coated culture dishes. Cultures were changed every 2 days and passaged every 5–7 days with StemPro Accutase Cell Dissociation Reagent (Gibco).

### 2.2 Mesenchymal stem cell differentiation

HiPSCs were differentiated into mesenchymal stem cells (MSCs) using protocols for differentiation published by Denis et al. (2011). Briefly, 1.10^4^ to 4.10^4^ hiPSCs/cm^2^ were seeded on 0.1% gelatin (Sigma)-coated dishes with knockout™ DMEM (Gibco) supplemented with 20% fetal bovine serum (Sigma), 1% non-essential amino acids (Gibco), 1% Glutamax™ (Gibco), 0.1% β-mercaptoethanol (Gibco), 1 mM ascorbic acid 2-phosphate (Sigma), and 10 ng/mL FGF2 (Tocris). The medium was changed every 2–3 days. MSCs were passaged with Trypsin-EDTA 1× (Gibco) each time they reached 80% confluence until they showed a characteristic fibroblastic spindle shape.

### 2.3 Adipocytic differentiation

MSCs were seeded at 20,000 cells/cm^2^ on 0.1% gelatin (Sigma)-coated dishes and cultured in the MSC medium previously described. At 80% of confluence, the medium was switched to StemMACS™ AdipoDiff Media, human (Miltenyi Biotec) for 10 days with medium changes every 2–3 days.

### 2.4 Osteogenic differentiation

MSC seeded at 10,000 cells/cm^2^ on Collagen I (100 μg/mL, Gibco)-coated plates were cultured in MSC medium, as previously described. At confluence, the medium was switched to osteogenic differentiation medium containing modified Eagle medium alpha (Gibco), 16.5% FBS, 100 nM dexamethasone (Sigma), 200 µM ascorbic acid 2-phosphate (Sigma), and 10 mM *β*-glycerophosphate (Sigma) for 14–20 days with medium changes every 2–3 days.

### 2.5 *NF1* (+/−) and *NF1* (−/−)-knockout isogenic hiPSC line generation by CRISPR/Cas9

Single-guide RNAs (sgRNAs) were designed using CRISPOR software (http://crispor.tefor.net) and synthesized by IDT™ (Integrated DNA Technologies). sgRNA1 (5′-TAACTGCGCAACCTTCTTTA-3′) and sgRNA2 (5′-GTTAGCAGTTATAAATAGCC-3′) target the exon 6 of *NF1* gene. Target-specific crispr RNA (crRNA) and trans-activating crispr RNA (tracrRNA) were synthesized by IDT. We electroporated 2.10^5^ control *NF1* (+/+) hiPSCs with 120 pmol of each sgRNA (obtained by duplexing crRNA and tracrRNA) and 100 pmol of *Streptococcus pyogenes* Cas9 protein (gift from Jean-Paul Concordet, MNHN/CNRS UMR 7196/INSERM U1154) using the P3 Primary Cell 4D-Nucleofector^®^ X Kit (Lonza) according to the manufacturer’s instructions. Transfected hiPSCs were then seeded in StemMACS™ iPS-Brew XF medium supplemented with Clone R^®^ (STEMCELL Technologies) in 24-well plates coated with Matrigel^®^ hESC qualified Matrix (Corning). After 48 h from transfection, the hiPSCs were harvested for high-resolution melting analysis and seeded back to obtain isolated clones using limiting dilution cloning in a 96-well plate. After 10–14 days of expansion, clones were analyzed for editing.

### 2.6 Identification of edited clones

The genomic DNA of single colonies was extracted using QuickExtract DNA extraction solution (Lucigen) following the manufacturer’s instructions. Targeted locus was amplified by PCR using MeltDoctor™ HRM Master Mix (Applied Biosystems) and primers (5′-TGC​TCT​GAG​TTG​TAT​TTG​TGT​TAA​C-3′, 5′-GAG​AGG​TTG​TAA​CTT​ACC​TTT​TCC​A-3′) according to the manufacturer’s instructions. PCR reactions were analyzed using a QuantStudio 12K Flex Real-Time PCR system (Applied Biosystems). The protocol was 95°C for 10 min, then 40 cycles of 95°C for 15 s and 60°C for 1 min, followed by 95°C for 10 s and 60°C for 1 min; then, the temperature was increased by 0.025°C/s until 95°C 15 s, followed by cooling to 60°C. Curves were analyzed using QuantStudio™ 12K Flex software. Clones showing editing were selected, and corresponding PCR products were sequenced by Sanger sequencing (Eurofins).

To ensure the purity of each edited clone, corresponding PCR products were subcloned using a TOPO™ TA Cloning™ Kit for Sequencing, with One Shot™ TOP10 Chemically Competent *E. coli* (Thermo Fisher Scientific) according to manufacturer’s instructions. At least 30 TA clones for each CRISPR cell line were analyzed by Sanger sequencing (Eurofins).

### 2.7 Surface antigen flow cytometry analysis

Expression of cell surface antigens on hiPSCs or mesenchymal stem cells (MSCs) was analyzed by flow cytometry. Cells were dissociated into single-cell suspension with Accutase (Invitrogen) and Tryple Express enzyme (Gibco) for hiPSCs and MSCs, respectively. Cells were incubated in PBS containing 2% fetal bovine serum with fluorescent dye-conjugated antibodies (listed in Supplementary Table S1) for 30 min on ice. Staining with the isotypic antibody was performed as a control. Analysis was performed on a MACSQuant^®^ system (Miltenyi Biotec). Data were analyzed using FlowJo software (FlowJo LLC).

### 2.8 Protein extraction and Western blot analysis

Total proteins were extracted in RIPA lysis buffer (Sigma) containing 1% protease inhibitor cocktail (Sigma) and 10% phosphatase inhibitor (Roche). Proteins were quantified using the Pierce BCA Protein assay kit (Thermo Fisher Scientific) and loaded using NuPAGE™ 3%–8% and Tris*-*acetate (Invitrogen). Total proteins were then transferred to PVDF membrane (Bio-Rad) with a Trans-Blot Turbo Transfert system (Bio-Rad), blocked with Odyssey blocking buffer (OBB) containing 0.1% Tween-20, and incubated overnight with the primary anti-neurofibromin antibody (abcam, ab17963, 1/1000) diluted in the OBB containing 0.1% Tween-20. The membrane was then incubated for 1 h with the corresponding IRDye secondary antibodies (LI-COR). Immunoreactive protein bands were revealed using an Odyssey CLx Imager (LI-COR) according to the manufacturer’s protocol. Anti-beta-Actin antibody (Santa Cruz Biotechnologies, sc4778, 1/10,000) or anti-vinculin (abcam, ab129002, 1/1000) was used to verify equal protein loading.

### 2.9 BODIPY staining

Adipocytic differentiation was monitored after staining lipid droplets. Briefly, cells were permeabilized with FCM permeabilization buffer (Santa Cruz Biotechnology), stained with BODIPY™ 493/503 (Invitrogen, 1/100 in PBS) and Hoechst (Invitrogen, 1/3000 in PBS) for 20 min, and rinsed.

### 2.10 Alkaline phosphatase staining and quantification

Osteogenic differentiation was tested on day 14 for staining of alkaline phosphatase activity. Cells were washed, fixed with ethanol 95%, and stained with the SIGMA*FAST*™ BCIP/NBT kit (Sigma) according to the manufacturer’s instructions. The quantification of ALP activity was performed using 1-Step™ PNPP Substrate Solution (Thermo Fisher Scientific). The results were normalized with the total viable cell number monitored with the CellTiter-Glo assay^®^ (Promega) according to the manufacturer’s instructions.

### 2.11 Alizarin red staining

Calcium deposition was detected with alizarin red staining solution (Merck Millipore) according to the manufacturer’s instructions. Briefly, after 20 days of culture, cells were washed, fixed with ethanol 70%, incubated for 20 min with alizarin red solution, and rinsed before microscopy observation.

### 2.12 RNA extraction and quantitative RT-PCR

Total RNAs were extracted using the RNeasy Micro/Mini Kit (Qiagen) and were reverse transcribed (RT) using the Superscript™ III reverse transcriptase kit (Invitrogen) with a mix of random hexamers and oligo dT according to the manufacturer’s instructions. Quantitative PCR (qPCR) was carried out with the primers listed in Supplementary Table S2 and Luminaris Color HiGreen qPCR Master Mix, low ROX (Thermo Fisher Scientific) using a QuantStudio 12K Flex Real-Time PCR system (Applied Biosystems). The amplification was performed using 95°C for 10 min, followed by 40 cycles at 95°C for 15 s and 60°C for 1 min. The relative gene expression level was calculated with the 2-delta delta Ct method and normalized to 18S expression.

### 2.13 Statistical analysis

All data were processed using GraphPad Prism 9^®^. Data were analyzed using the non-parametric Mann–Whitney test for side-by-side comparison. *p-*values ≤0.05 were considered significant.

## 3 Results

### 3.1 Generation and characterization of *NF1* (+/−) and (−/−) isogenic hiPSCs using CRISPR/Cas9

**FIGURE 1:**
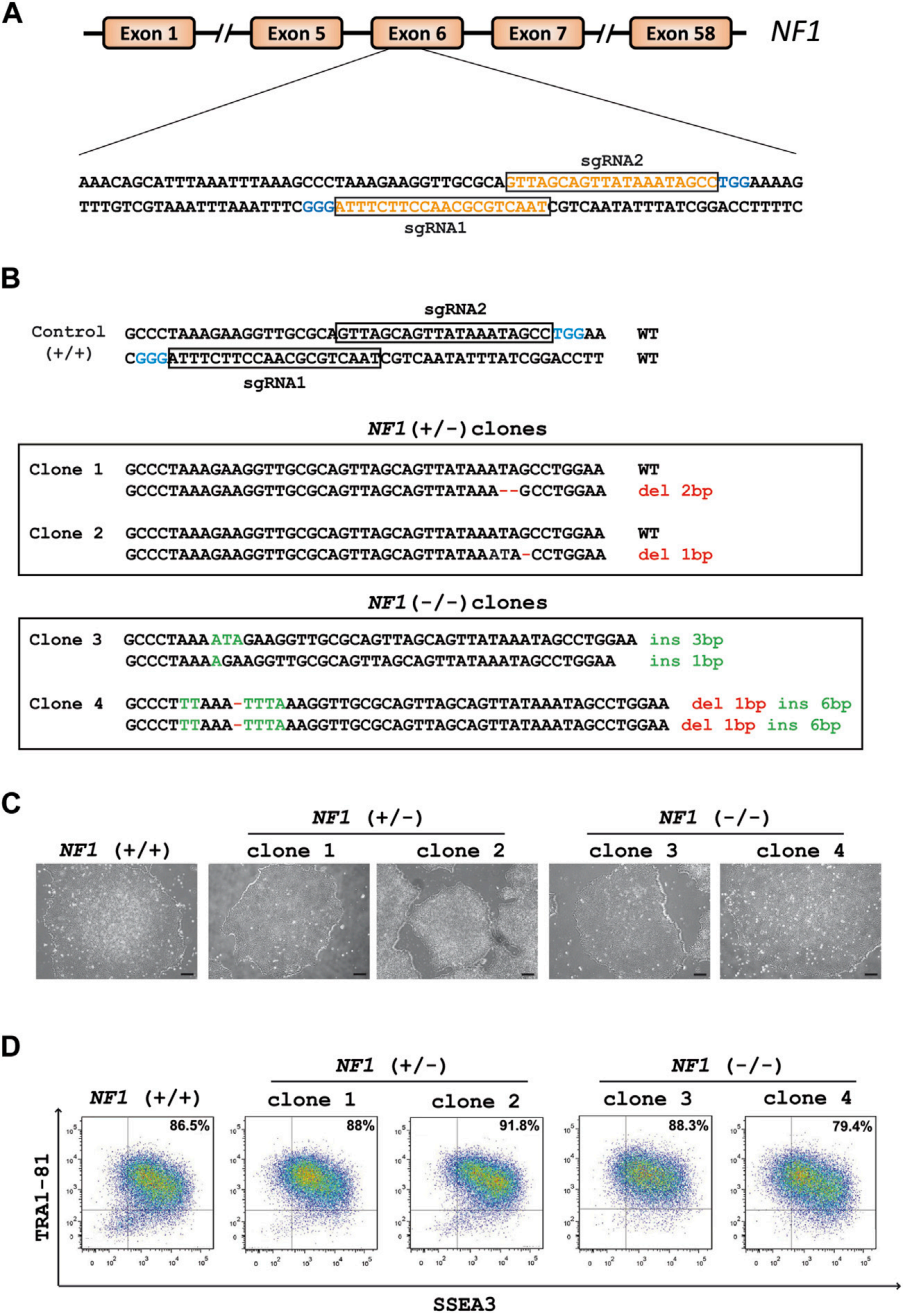
Generation of heterozygous (+/−) and homozygous (−/−) *NF1* isogenic hiPSCs by Crispr/Cas9. **(A)** Schematic representation of the *NF1* gene. SgRNA1 and sgRNA2 sequences are shown in orange and target exon 6 of the *NF1* gene. PAM sequences are in blue. **(B)** Sequences of the targeting region in human *NF1* gene. Insertions (ins, green letters) and deletions (del, red letters) are indicated. The sequence of *NF1* (+/+) is indicated above. *NF1* (+/−) clones 1 and 2 harbor one wild-type (WT) allele and a deletion of two or one base pairs (bp) on the edited allele, respectively. *NF1* (−/−) clone 3 carries one allele with an insertion of 1 bp and another allele with an insertion of 3 bp. *NF1* (−/−) clone 4 is homozygous for an insertion of 6 bp and deletion of 1 bp. **(C)** Phase contrast microscopic images of *NF1* (+/+), *NF1* (+/−), and *NF1* (−/−) hiPSCs. Scale bar: 200 µm. **(D)** Characterization of the expression of pluripotency markers SSEA3 and TRA1-81 by flow cytometry in control *NF1* (+/+), *NF1* (+/−), and *NF1* (−/−) hiPSCs. Staining with isotypic antibody was performed as a control.

CRISPR/Cas9 technology was used to generate *NF1* (+/−) and (−/−) isogenic hiPSCs. Two single-guide RNAs (sgRNA1 and sgRNA2) were designed to target exon 6 of the *NF1* gene (Figure 1A). HiPSCs were transfected with each sgRNA complexed with Cas9 protein. The global editing activity of Cas9 protein in the bulk of transfected cells using T7 endonuclease assay was approximately 38% and 5% for sgRNA1 and sgRNA2, respectively (Supplementary Figure S1A). A total of 24 colonies per condition were isolated from the pool of transfected cells using a limiting dilution procedure. The genomic DNA of each clone was examined by high-resolution melting (HRM). The analysis showed that 88% and 42% of the cells were edited for sgRNA1 and sgRNA2, respectively. Additional molecular analyses were carried out on the selected clones. Amplicon sequencing generated by PCR revealed that both sgRNA produce several INDELs, including deletion, insertion, or substitution (Supplementary Figure S1B). Sequencing analysis indicated that sgRNA1 and sgRNA2 led to a bi-allelic and a mono-allelic edition, respectively, in all generated clones. Among the edited clones with the sgRNA2 and sgRNA1, respectively, two edited hiPSC clones with mono-allelic mutations (*NF1* (+/−) clone 1 and *NF1* (+/−) clone 2) and two clones with bi-allelic mutations (*NF1* (−/−) clone 3 and *NF1* (−/−) clone 4 were used for further characterization. Sequencing showed that *NF1* (+/−) clones 1 and 2 carry an unmodified allele and a deletion of two or one base pairs (bp) on the other allele, respectively (Figure 1B, Supplementary Figures S2A, S2B). *NF1* (−/−) clone 3 carries one allele with an insertion of 1 bp and another allele with an insertion of 3 bp (Figure 1B, Supplementary Figure S2C). *NF1* (−/−) clone 4 is homozygous for an insertion of 6 bp and deletion of 1 bp (Figure 1B, Supplementary Figure S2D). In addition, each mutation resulted in a frameshift mutation in the *NF1* gene, which led to a premature stop codon and a truncated protein (approximately 23 KDa instead of 319 KDa).

Quality control experiments were then performed for each selected edited hiPSC clone as well as their parental hiPSCs. The typical morphology of hiPSCs, which involves highly packed cells and clear borders, was observed in all hiPSC clones (Figure 1C). The expression of proteins related to pluripotency was evaluated through flow cytometry using surface markers TRA1-81 and SSEA3 (Figure 1D). Genomic integrity was also confirmed by SNP genotyping (Supplementary Figure S3). To detect off-target activity (OFT), the eight most likely off-target sites predicted by the CRISPOR tool (https://crispor.tefor.net/) were analyzed (Supplementary Table S3). Gene editing did not cause any mutations, except for *NF1* (+/−) clone 2, which presents a mutation in an intron of the TMEM163 gene (OFT site 1 of sgRNA2) (Supplementary Figure S4A, S4B). However, using quantitative RT-PCR experiments, we demonstrated that the TMEM163 gene is not expressed in any cell types used in this study (Supplementary Figure S4C). Collectively, we successfully generated two *NF1* (+/−) and two *NF1* (−/−) isogenic hiPSC clones, which maintain a pluripotent phenotype, essential for an efficient differentiation.

### 3.2 *NF1* (+/−) and (−/−) isogenic hiPSCs possess normal capabilities to generate mesenchymal stem cells

**FIGURE 2:**
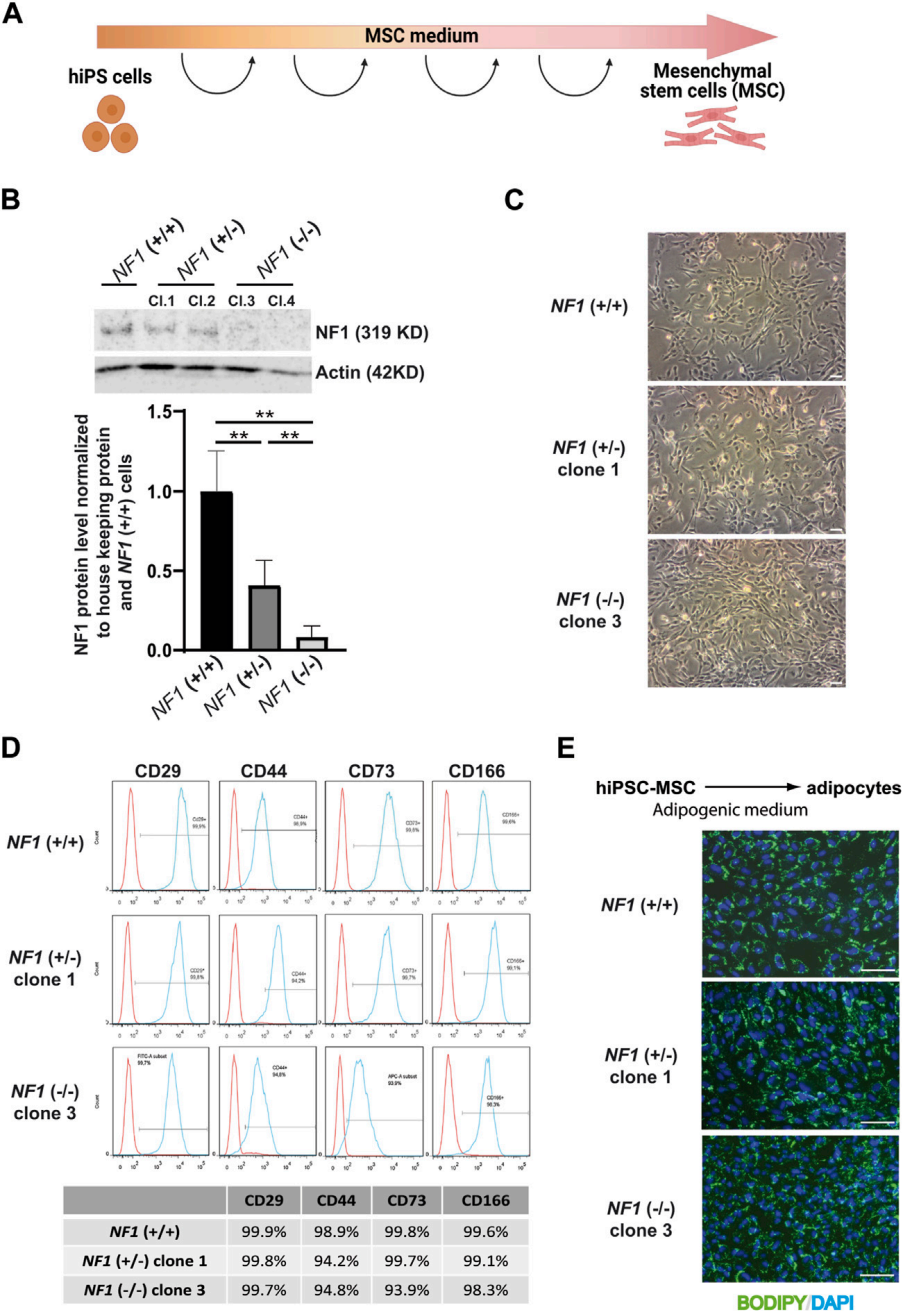
Characterization of mesenchymal stem cells derived from *NF1* (+/−) and *NF1* (−/−) isogenic and their parental *NF1* (+/+) hiPSCs. **(A)** Schematic representation of MSC differentiation protocol. Created with Biorender.com. **(B)** Western blot analysis of NF1 protein expression by *NF1* (+/+), *NF1* (+/−), and *NF1* (−/−) hiPSC-MSCs. Actin served as loading control. Densitometry measurement of protein levels relative to control *NF1* (+/+). Data are represented as mean ± standard deviation (SD) (*n* = 3 independent experiments with at least three hiPSC-MSC differentiations for each condition, mean of two clones for (+/−) and (−/−)). Statistical analyses are based on a Mann‒Whitney nonparametric test for side by side comparison. **p < 0.01. **(C)** Representative phase contrast images in *NF1* (+/+), *NF1* (+/−) clone 1, and *NF1* (−/−) clone 3 MSCs generated from hiPSCs. Scale bar: 100 µm. **(D)** Representative flow cytometry analysis of CD29, CD44, CD73, and CD166 MSC-specific markers in *NF1* (+/+), *NF1* (+/−) clone 1, and *NF1* (−/−) clone 3 hiPSC-derived MSCs. Isotypic staining control peaks are shown in red. **(E)** Representative images of DAPI nuclear staining (blue) and BODIPY staining (green) performed after 10 days of adipogenic differentiation from *NF1* (+/+), *NF1* (+/−) clone 1, and *NF1* (−/−) clone 3 hiPSC-MSCs. Scale bar: 100 µm. Results of *NF1* (+/−) clone 2 and *NF1* (−/−) clone 4 are presented in Supplementary Figure S4.

The *NF1* (+/−) and *NF1* (−/−) hiPSCs and their control *NF1* (+/+) hiPSCs were then differentiated into a homogenous population of mesenchymal stem cells (MSCs) capable of differentiating into various lineages, including the osteoblastic lineage. Briefly, hiPSCs were cultured in MSC differentiation medium and passaged each time they reached confluence until they showed a characteristic fibroblastic spindle shape (Figure 2A). We first verified the expression of neurofibromin at mRNA and protein levels in hiPSC-derived MSCs (hiPSC-MSCs). RT-PCR experiments showed that expression of NF1 mutated mRNA was decreased in comparison to NF1 control mRNA (Supplementary Figure S5A). Western blotting demonstrated that the expression of full-length neurofibromin was significantly reduced by half in the *NF1* (+/−) hiPSC-MSCs compared to the *NF1* (+/+) hiPSC-MSCs (Figure 2B). No expression of full-length neurofibromin was detectable in the *NF1* (−/−) hiPSC-MSCs (Figure 2B). To support the relevance of using human isogenic iPSCs to investigate the etiology of NF1, we assessed the signaling protein MAPK (mitogen-activated protein kinase), which has been reported to be altered by the loss of NF1. Our data suggest that NF1 loss in our system leads to an increase in pERK levels in the *NF1* (+/−) and *NF1* (−/−) hiPSC-MSCs compared to control *NF1*(+/+) (Supplementary Figure S5B). For all the following analyses, the results for *NF1* (+/−) clone 1 and *NF1* (−/−) clone 3 are shown in the main figures and are confirmed in other clones (*NF1* (+/−) clone 2 and *NF1* (−/−) clone 4) in the Supplementary Figures. Control *NF1* (+/+), *NF1* (+/−), and *NF1* (−/−) hiPSC-MSCs were able to grow under standard culture conditions and displayed a normal fibroblastic morphology (Figure 2C, Supplementary Figure S5C). The generated hiPSC-MSCs were then characterized. MSCs are defined by their ability to express cell surface markers including CD29, CD44, CD73, and CD166 and to differentiate into adipocytes (Dominici et al., 2006). Cell surface protein expression analysis by flow cytometry showed that the expression of CD29, CD44, CD73, and CD166 was more than 92% in *NF1* (+/−) and *NF1* (−/−) hiPSC-MSCs in the same manner as *NF1* (+/+) hiPSC-MSCs (Figure 2D, Supplementary Figure S5D). Finally, to assess the adipocytic differentiation capacity of the *NF1* (+/−) and (−/−) cell lines, hiPSC-MSCs were grown in adipogenic differentiation medium for 10 days. Lipid accumulation was measured by BODIPY staining. Data demonstrated similar intense staining in both lines, as observed in the *NF1* (+/+) hiPSC-MSCs with lipid droplets within the cytoplasm of all cells (Figure 2E, Supplementary Figure S5E). Moreover, SNP genotyping showed no alteration of the genomic integrity of *NF1* (+/−) and *NF1* (−/−) hiPSC-MSCs, as also observed in *NF1* (+/+) lines (Supplementary Figure S6). Altogether, these data indicate that partial or total loss of neurofibromin expression does not alter the potential of *NF1* (+/−) and (−/−) hiPSCs to differentiate into MSC and then into adipocytes.

### 3.3 *NF1* (+/−) and *NF1* (−/−) isogenic hiPSC-MSCs exhibit a defect of osteogenic differentiation capacity

**FIGURE 3:**
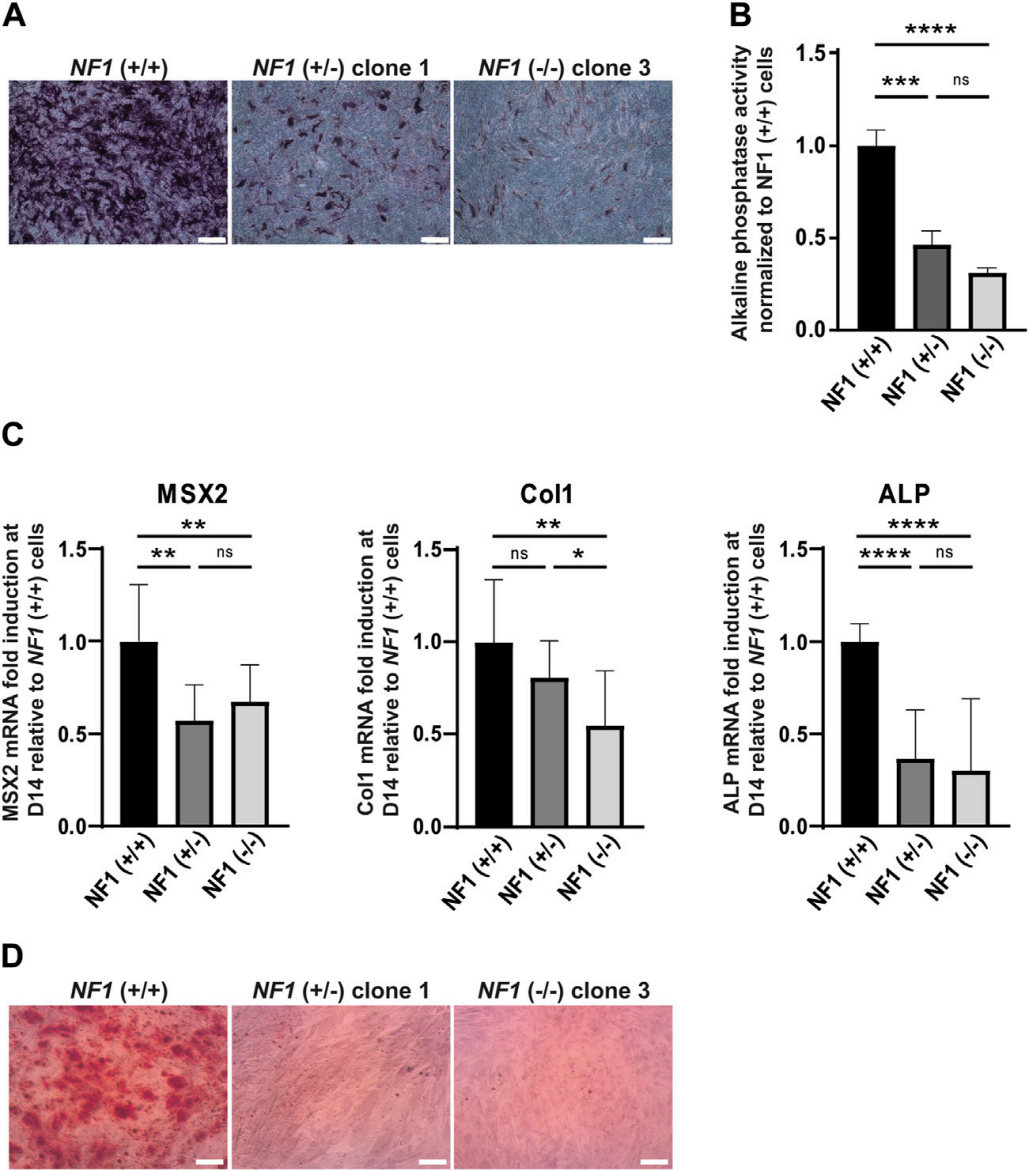
Defect in osteogenic differentiation of mesenchymal stem cells derived from *NF1* (+/−) and *NF1* (−/−) isogenic hiPSCs. **(A)** Representative images of alkaline phosphatase (ALP) staining of *NF1* (+/+), *NF1* (+/−) clone 1, and *NF1* (−/−) clone 3 hiPSC-MSCs performed after 14 days of culture in the osteogenic differentiation medium. Scale bar: 50 µm **(B)** ALP activity level of *NF1* (+/+), *NF1* (+/−), and *NF1* (−/−) hiPSC-MSCs using pNPP assay performed after 14 days of culture in osteogenic differentiation medium. Data correspond to mean±SD after normalization on the mean value obtained with *NF1* (+/+) hiPSC-MSCs (*n* = 3 independent experiments with at least three hiPSC-MSC differentiations for each condition, mean of two clones for *NF1* (+/−) and *NF1* (−/−)). Statistical analyses are based on a Mann‒Whitney nonparametric test for side-by-side comparison. ***p < 0.001 and ****p < 0.0001; ns: not significant. **(C)** Analysis of osteoblast-specific marker expression by quantitative RT-PCR in *NF1* (+/+), *NF1* (+/−), and *NF1* (−/−) hiPSC-MSCs after 14 days of osteogenic differentiation. Transcript level of Msh homeobox 2 (MSX2), collagen 1 (Col1), and alkaline phosphatase (ALP) are normalized to 18S RNA. Histograms represent mRNA fold induction between days 14 (D14) and 0 (D0) and are expressed relative to control *NF1* (+/+) cells. Data obtained from at least three independent experiments (two clones per condition) are represented as mean±SD. Statistical analyses are based on a Mann‒Whitney nonparametric test for side-by-side comparison. *p < 0.05, **p < 0.01, and ****p < 0.0001; ns: not significant. **(D)** Representative images of calcium deposition after alizarin red staining performed at 20 days of osteogenic differentiation of *NF1* (+/+), *NF1* (+/−) clone 1, and *NF1* (−/−) clone 3 hiPSC-MSCs. Scale bar: 50 µm. Results of *NF1* (+/−) clone 2 and *NF1* (−/−) clone 4 are presented in Supplementary Figure S4.

In addition to their ability to differentiate into adipocytes, mesenchymal stem cells also have the capacity to differentiate into the osteoblastic lineage. To investigate the impact of *NF1* inactivation on osteoblastic differentiation, *NF1* (+/−) and *NF1* (−/−) hiPSC-MSCs were cultured in osteogenic differentiation medium. After 14 days of culture, staining of alkaline phosphatase (ALP), an initial marker of osteoblast differentiation, was performed. As shown in Figure 3A and in Supplementary Figure S7A, ALP activity staining was less intense in *NF1* (+/−) and *NF1* (−/−) cells than in *NF1* (+/+) cells. To accurately measure the decrease in ALP activity in *NF1* (+/−) and (−/−) cells, a quantitative assay using a para-nitrophénylphosphate (pNPP) colorimetric assay was secondarily conducted. According to our finding, *NF1* (+/−) and *NF1* (−/−) hiPSC-MSCs showed a 2.2-fold and 3.2-fold decrease, respectively, compared to *NF1* (+/+) cells (Figure 3B). In addition, the expression of osteogenic markers, such as MSX2, Col1A, and ALP, was quantitatively assessed by RT-qPCR on days 0 and 14 after the initiation of osteoblastic differentiation. As shown in Figure 3C, the induction fold of MSX2, ColI, and ALP during osteoblastic differentiation in *NF1* (+/−) and *NF1* (−/−) cells decreased compared to *NF1* (+/+) cells, revealing a defective early osteoblastic differentiation. Moreover, when both *NF1* alleles were inactivated, we observed a general downward trend in phosphatase alkaline activity (Figures 3A–C). Calcium deposits occur in the matrix of the osteoblasts in the later stage of osteogenic differentiation. Therefore, the calcification of cells was visualized using alizarin red staining solution (Figure 3D, Supplementary Figure S7B). After 20 days of differentiation, both *NF1* (+/−) and *NF1* (−/−) cells generated from hiPSC-MSCs showed decreased calcium deposit compared to *NF1* (+/+) cells, suggesting that *NF1* (+/−) and *NF1* (−/−) cells have a reduced ability to form a mineralized matrix (Figure 3D, Supplementary Figure S7B). Together, these data demonstrate an osteoblastic differentiation defect in *NF1* (+/−) and *NF1* (−/−) cells. Our results also reveal that the inactivation of one *NF1* allele is sufficient to alter osteoblastic differentiation and suggest that a bi-allelic inactivation of *NF1* may cause a more severe phenotype.

### 3.4 Validation of the osteoblast differentiation defect by using hiPSC-MSCs derived from NF1 patients

**FIGURE 4:**
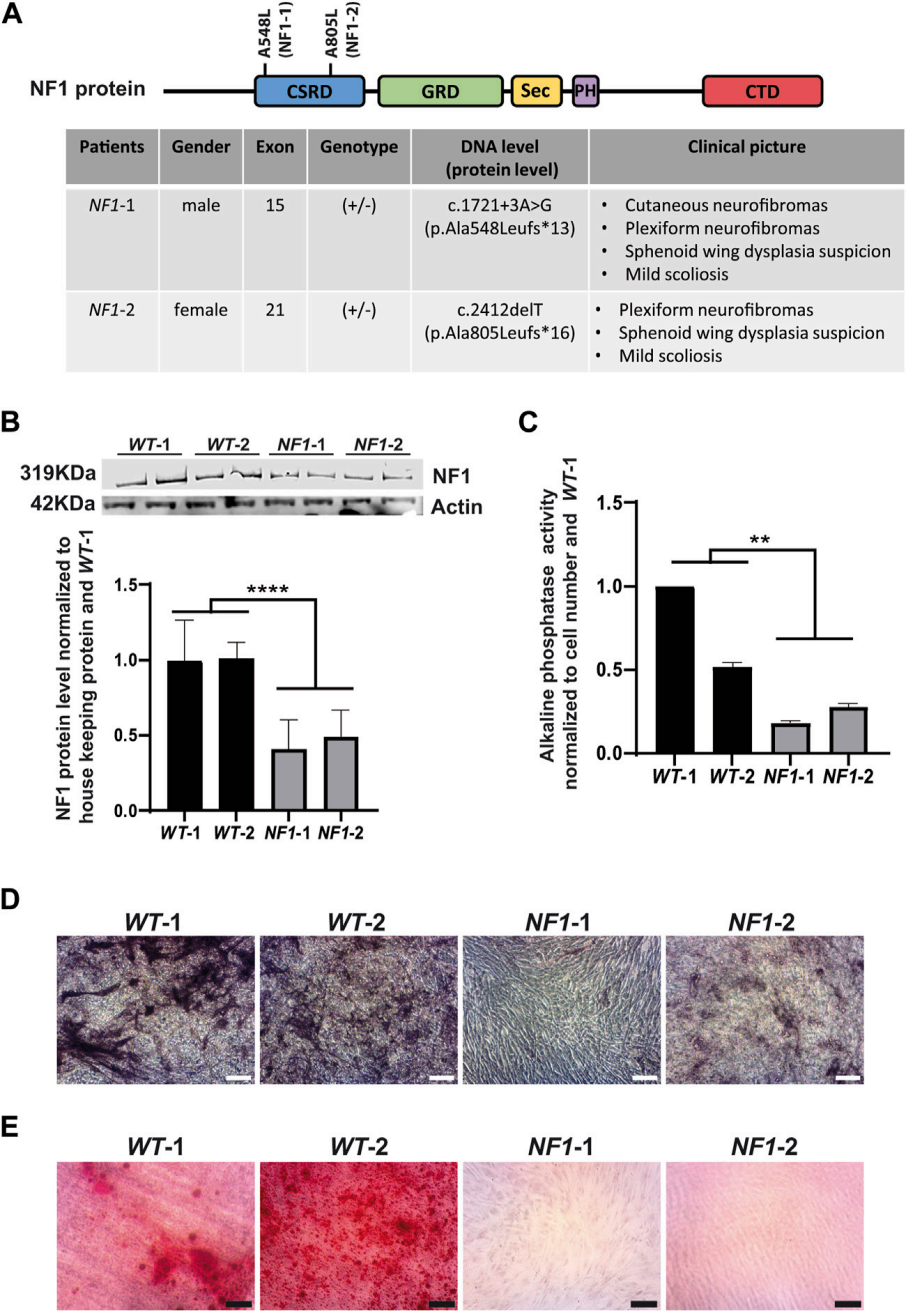
Defect in osteogenic differentiation of mesenchymal stem cells derived from NF1 patient hiPSCs. **(A)** Schematic representation of neurofibromin protein and table of NF1 patient mutations. CSRD (cysteine–serine-rich domain) is shown in blue, GRD (GTPase-activating protein-related domain) in green, Sec (Sec14 homologous domain) in yellow, PH (pleckstrin homologous domain) in purple, and CTD (carboxy-terminal domain) in red. The position of genetic variations detected in the *NF1* gene from each patient was identified in the domain CSRD, as shown in the scheme of NF1 protein. The table specifies patient gender, mutation location, zygosity, cDNA, protein consequences, and clinical pictures. **(B)** Western blot analysis of the NF1 protein expression level in control (*WT*-1 and *WT*-2) and NF1 patient (*NF1*-1 and *NF1*-2) hiPSC-MSCs. Actin served as loading control. Densitometry measurement of protein levels was relative to control *NF1* (+/+) *WT-1*. Data are represented as mean± SD of at least three independent experiments. Statistical tests were performed by comparing averages of data obtained with control cells (*WT-1* and *WT-2*) with averages of the data obtained for NF1 lines (*NF-1* and *NF1-2*), using a Mann‒Whitney nonparametric test. ****p < 0.0001. **(C)** ALP activity level in control (*WT*-1 and *WT*-2) and NF1 patient (*NF1*-1 and *NF1*-2) hiPSC-MSCs performed after 14 days of culture in osteogenic differentiation medium. Data obtained from three independent experiments for each cell line are represented as mean±SD after normalization on cell number and on the value obtained with *WT*-1 hiPSCs-MSCs. Statistical analyses are based on a Mann‒Whitney nonparametric test by comparing the mean of control cells to the mean of NF1 cells, **p < 0.01. **(D)** Alkaline phosphatase staining of control (*WT*-1 and *WT*-2) and NF1 patient (*NF1*-1 and *NF1*-2) hiPSC-MSCs performed after 14 days of culture in osteogenic differentiation medium; scale bar: 50 µm. **(E)** Calcium deposition with alizarin red staining performed after 20 days of osteogenic differentiation; scale bar: 50 µm.

Two hiPSC lines were reprogrammed from primary fibroblasts of NF1 patients (*NF1*-1 and *NF1*-2) to validate the relevance of using of the *NF1* isogenic hiPSCs generated by CRISPR/Cas9. *NF1*-1 and *NF1*-2 patients carry heterozygous mutations in the *NF1* gene, leading to the formation of a premature stop codon, resulting in the expression of a truncated neurofibromin (Figure 4A). Both were diagnosed with bone complications, such as sphenoid wing dysplasia and scoliosis (Figure 4A). The two *NF1* hiPSC lines (*NF1*-1 and *NF1*-2), the parental (+/+) control hiPSC line (*WT*-1) used previously and another control line (*WT-2*), were compared and differentiated into MSCs. According to Western blot analysis, expression of full-length neurofibromin was reduced by half in *NF1*-1 and *NF1*-2 hiPSC-MSCs compared to *WT*-1 and *WT*-2 hiPSC-MSCs (Figure 4B). To evaluate the potential of hiPSC-MSCs derived from NF1 patients to differentiate into the osteoblastic lineage, hiPSC-MSCs were cultured in osteogenic differentiation medium. After 14 days of differentiation, *NF1*-1 and *NF1*-2 cells harbored decreased alkaline phosphatase activity compared to *WT*-1 and *WT*-2 cells (Figures 4C,D). Furthermore, alizarin red staining solution showed a significant decrease in calcium deposition in cells derived from NF1 patients in comparison to control *NF1* (+/+) (Figure 4E). Collectively, these findings demonstrate that hiPSC-MSCs derived from NF1 patients reproduce the defect in osteoblastic lineage, as demonstrated in the *NF1* (+/−) and *NF1* (−/−) isogenic hiPSC CRISPR engineering, suggesting that *NF1* (+/−) and (−/−) isogenic hiPSCs could complement the currently available NF1 models and be used to better understand the osteogenic abnormalities observed in NF1 patients.

## 4 Discussion

Up to 50% of patients with NF1 develop characteristic osseous lesions (Elefteriou et al., 2009; Ma et al., 2020). The role of neurofibromin in the pathogenesis of bone abnormalities in patients with NF1 is not fully understood. To investigate how the level of NF1 expression contributes to osseous defects, we generated two heterozygous and two homozygous *NF1* isogenic hiPSCs obtained from one parental control *NF1* (+/+) hiPSC line using CRISPR/Cas9-based gene editing.

While protocols have been developed to differentiate hiPSCs into osteoblasts, the impact of NF1 loss in osteoblastic differentiation has not yet been investigated (Csobonyeiova, 2017; Diaz-Hernandez et al., 2023); hiPSCs have never been used to model osseous defects found in NF1 patients. However, several studies have demonstrated the potential of human pluripotent stem cells to reproduce other phenotypes associated with NF1. Our group has already shown the potential offered by human-derived embryonic stem cells to faithfully reproduce and explore the physiopathological mechanisms involved in the pigment cell-related manifestations of NF1 (Allouche et al., 2015). Human iPSCs have also been widely used to model NF1-associated brain and nerve pathology (Wegscheid et al., 2018). In these studies, hiPSCs were reprogrammed from cells extracted from NF1 patient biopsies (typically dermal fibroblasts or fibroblasts/Schwann cells derived from neurofibromas). However, the generation of isogenic *NF1* (+/−) and (−/−) hiPSCs that share the same genetic and epigenetic background generated by gene editing has not yet been achieved.

To study the osseous manifestations associated with NF1 and to rule out contributing factors such as genetic and epigenetic variations or gender (Liang and Zhang, 2013; Volpato and Webber, 2020), we generated two *NF1* (+/−) and two *NF1* (−/−) isogenic hiPSCs using the CRISPR-Cas9 system. Our work follows a recent study in which Carriò et al. successfully generated isogenic *NF1* (+/−) and *NF1* (−/−) hiPSCs, both isolated from fibroblasts and Schwann cells, respectively, from the same plexiform neurofibroma (Carrió et al., 2019). The same team used this iPSC-based *NF1* (−/−) model to form neurofibroma tumors when engrafted in the sciatic nerve of nude mice, demonstrating the potential of this model to capture the genomic status of the initial tumors and their ability to retain their features (Mazuelas et al., 2022). In our model, *NF1* (+/−) and (−/−) isogenic hiPSCs, which, respectively, exhibit reduced or absent expression of neurofibromin, were successfully differentiated into MSCs. MSC derived from *NF1* (+/−) and *NF1* (−/−) isogenic hiPSCs exhibit impaired osteoblastic differentiation, as evidenced by decreased mRNA expression of osteoblastic markers, decreased alkaline phosphatase activity, and reduced osteoblast mineralization. Moreover, hiPSC-MSCs derived from NF1 patients also reproduce the defect in osteoblast differentiation and mineralization. These findings are in line with previous studies that demonstrated that neurofibromin is important for regulating the osteoblast differentiation of murine *NF1* (+/−) or (−/−) MSCs (Wu, 2006; Kolanczyk et al., 2007; Sharma et al., 2013; Li et al., 2019). Other research has also confirmed an osteoblast differentiation defect in human MSC isolated from NF1 patients diagnosed with bone abnormalities (Leskelä et al., 2009; Sharma et al., 2013). In addition, the defect of mineralization has also been observed in different mouse models, in which *NF1* is specifically inactivated in osteoblasts (Elefteriou et al., 2006; Kühnisch et al., 2014; Ahmed et al., 2022). Interestingly, the single-cell sequencing trajectory analysis has been used to explore the genotype-specific osteogenic potential of a mixed population of human bone marrow stromal cells, including cells with both *NF1* homozygous and *NF1* heterozygous mutations. They highlighted a shared molecular pathology from the fracture site of a patient with NF1, indicating potential targeted therapies for NF1 pseudarthrosis treatment (Paria et al., 2023). In our model, we show that *NF1* (+/−) and (−/−) isogenic hiPSCs recapitulate the defect in osteoblast differentiation and the reduced bone mineral density associated with NF1 patients (Ferrara et al., 2022). Several studies have shown by genotypic analysis a localized loss of heterozygosity of the *NF1* gene in pseudarthrosis or scoliosis tissues, suggesting that biallelic inactivation is necessary to develop bone abnormalities (Stevenson et al., 2006; Lee et al., 2012; Paria et al., 2014; Margraf et al., 2019). In our study, the comparison of isogenic *NF1* (+/−) and control *NF1* (+/+) lines which differ only by mutation of one *NF1* allele, showed a defect in osteoblastic, differentiation in *NF1* (+/−) lines. This result provides a direct correlation between the genotype and phenotype and demonstrates that *NF1* bi-allelic inactivation is not necessarily required to promote the osteoblastic differentiation defect. In addition, although we observed a general tendency for a more pronounced differentiation defect in *NF1* (−/−) than *NF1* (+/−) lines, our results did not clearly show a significant difference when both *NF1* alleles were inactivated. More investigations are needed to clarify the consequences of the loss of heterozygosity observed in NF1 patients, which could have different effects depending on the cell microenvironment. Furthermore, it has been recently demonstrated that, besides *NF1* invalidation, additional genetic causes or modifier genes could be involved, which could explain the phenotypic variability observed in patients (Wang et al., 2021). This suggests that additional factors/modifier genes may also contribute to osseous defects, as shown by Pacot et al., in neurofibromas associated with NF1 (Pacot et al., 2023). In addition, Anastasaki et al. have also demonstrated that the use of isogenic hiPSCs with different heterozygous germline *NF1* gene mutations has varying effects on human central nervous system cells (Anastasaki et al., 2020). In this study, we generated isogenic cell lines on one single genetic background. Hence, engineering other isogenic hiPSC lines with mutations that target regions other than exon 6 of the *NF1* gene or in another genetic context will be essential to determine how various mono-allelic or bi-allelic *NF1* mutations affect osseous abnormalities.

Bone homeostasis is maintained by a balance between bone formation by osteoblasts and bone resorption by osteoclasts. Previous mouse studies in NF1 haploinsufficient mice (Yang et al., 2006; Alanne et al., 2012; Sharma et al., 2013) and from human NF1 patients (Yang et al., 2006; Heervä, 2010; Stevenson et al., 2011) have reported that haploinsufficiency of NF1 results in a generalized osteoclast gain in function, which could contribute to increased bone resorption. However, Stevenson et al. (2011) have demonstrated that increased osteoclast formation *in vitro* cannot predict NF1 skeletal phenotypes, suggesting also that additional modifications, such as secondary genetic mutations or modifier genes, are also required for the development of skeletal abnormalities in NF1 (Stevenson et al., 2011). Recently, iPSC lines have been successfully differentiated into osteoclasts (Chen, 2020; Rössler et al., 2021). Thus, our *NF1* isogenic hiPSC model is interesting for studying how bi-allelic inactivation affects osteoclast function and how osteoclasts influence bone defects.

Human iPSCs have been widely used in various 2D disease modeling studies. The emergence of organoids in cellular systems has recently presented the possibility of mimicking the interactions between the different cell types in a closer physiological system (Silva-Pedrosa et al., 2023). As NF1 implies gain-in osteoclast functions and impaired osteoblast differentiation, the use of 3D co-culture models using isogenic hiPSC-derived osteoclasts together with isogenic hiPSC-derived osteoblasts will be valuable for examining the interactions between cell types and for understanding the progression of skeletal defects in NF1 patients. Furthermore, the 3D coculture system could also be used in a high-throughput screening approach to identify molecules that could rescue osteoblast and osteoclast activity.

Collectively, our data demonstrate the potential and importance of developing an isogenic *NF1* iPSC CRISPR engineering model with a fixed genetic background, which may help the genotype–phenotype correlation. This model offers the possibility of studying and validating molecular mechanisms associated with NF1.

## Data Availability

The original contributions presented in the study are included in the article/Supplementary Material; further inquiries can be directed to the corresponding authors.
