# Supplementary material for: Generation of heterozygous and homozygous NF1 lines from human-induced pluripotent stem cells using CRISPR/Cas9 to investigate bone defects associated with neurofibromatosis type 1
Source: Front Cell Dev Biol. 2024 Feb 28;12:1359561. doi: 10.3389/fcell.2024.1359561 (PMC10935092; doi:10.3389/fcell.2024.1359561)
Supplement: Supplementary file 1 [file DataSheet1.pdf]

Supplementary Material

Generation of heterozygous and homozygous NF1 lines from human induced pluripotent stem cells using CRISPR/Cas9 to investigate bone defects associated to neurofibromatosis type 1

Annabelle Darle[1], Thibault Mahiet[1], Deborah Aubin[2,3,6], Manon Doyen[2,3], Lina El Kassar[1], Beatrice Parfait[4,5], Gilles Lemaitre[2,3], Christine Baldeschi[2,3], Jennifer Allouche[2,3*†] and Nathalie Holic[2,3*†]

[1] Centre d'Etude des Cellules Souches, 91100 Corbeil-Essonnes, France
[2] Université Paris-Saclay, Université d'Evry, U861, 91100 Corbeil-Essonnes, France
[3] INSERM U861, I-Stem, AFM, Institute for Stem Cell Therapy and Exploration of Monogenic Diseases, 91100 Corbeil-Essonnes, France.
[4] Service de Génétique et Biologie Moléculaires, Hôpital Cochin, Hôpitaux Universitaires Paris Centre, Assistance Publique-Hôpitaux de Paris (AP-HP), Paris, France.
[5] EA7331, Faculté de Pharmacie de Paris, Université Paris Descartes, Paris, France
[6] Phenocell SAS, Grasse, France

[†] Last authorship

*** Correspondence:** Corresponding Authors: nholic@istem.fr and jallouche@istem.fr

1 Supplementary Material

T7 endonuclease I assay

48 hours post-transfection, genomic DNA was extracted from the high density plate using the QuickExtract DNA extraction solution (Lucigen) following the manufacturer's instructions. A 530 bp fragment around the targeted locus was amplified by PCR using Phusion® High-Fidelity DNA Polymerase (New England Biolabs) and primers (GCAGACAACTATCGAGTTTTGGG, CCAGCCATATCAGTCTGTGGG) according to the manufacturer's instructions. Amplicons were subjected to T7 endonuclease I (New England Biolabs) following the manufacturer's instructions. Products obtained after digestion were then analyzed using the bioanalyzer 2100 (Agilent). The cleavage efficiency was calculated as the ratio of cleaved amplicons to the total (uncleaved and cleaved) amplicons.

Off-target analysis

We used the online tool CRISPOR (https://crispor.tefor.net/) to identify the eight most-likely off-target regions that could be targeted by sgRNA1 and sgRNA2 (Supplementary Table S3). The region surrounding each off-target site was amplified by PCR using specific primers listed in Supplementary Table S4. PCR reactions were performed using the Phusion High-Fidelity DNA Polymerase Kit (Thermofisher Scientific) with 200ng genomic DNA, 30 cycles of 10sec at 98 °C, 30sec at 60-66 °C and 15sec at 72 °C, with a final 5min extension at 72 °C. PCR products were then sequenced by Sanger sequencing (Genwiz) and compared to the unedited hiPSCs.

SNP genotyping

High quality genomic DNA was obtained from 5.10^6 cells using QIAcube™ workstation using QIAamp® DNA Blood Mini Kit DNA (Qiagen). Genomic DNA hybridation was achieved on Infinium Core-24v1-2 BeadChip (Illumina). Data were analyzed with GenomeStudio v2.0.5 software (Illumina).

MAPK western blot analysis

Total proteins were extracted in RIPA lysis buffer (Sigma) supplemented with antiproteases/antiphosphatases, and quantified using the Pierce BCA Protein assay kit (Thermofisher Scientific). SDS-PAGE was performed using NuPAGE Novex 4-12% (BioRad). Primary antibody incubation was followed by incubation with specific horseradish peroxidase–conjugated secondary antibodies. Primary antibodies include pMAPK (Cell Signaling cat#9101, 1/1,000) and total MAPK (Cell Signaling cat#9102, 1/1,000). Immunoreactive bands were revealed using Amersham ECL Plus Western blotting detection reagents (GE Healthcare). Quantification was performed using using an Odyssey CLx Imager (LI-COR).

2 Supplementary Figures and Tables

2.1 Supplementary Figures

Supplementary Figure S1

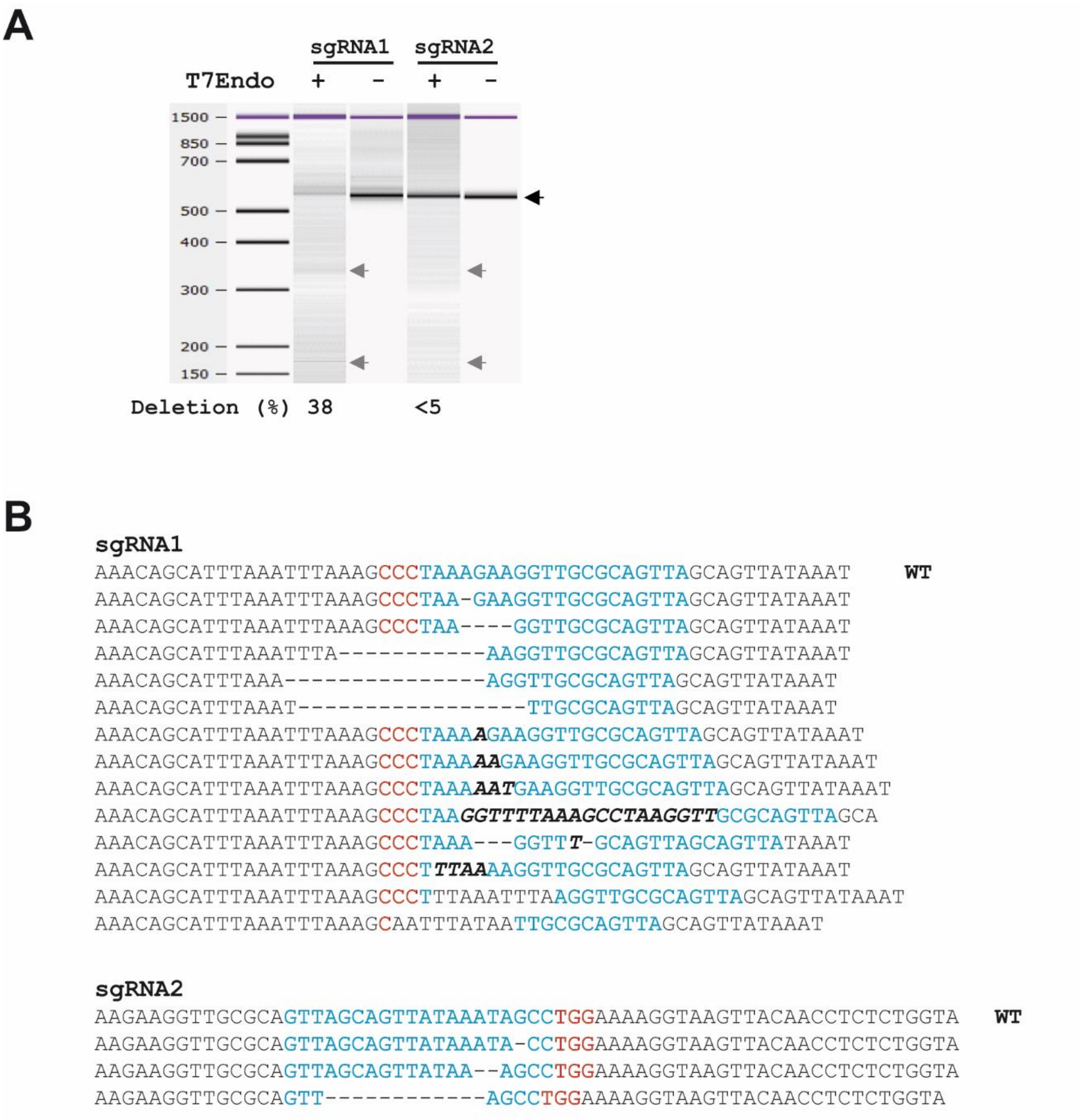

Supplementary Figure S1. Evaluation of Crisp-Cas9 gene editing efficiency. **(A)** Evaluation of DNA editing activity using T7 endonuclease I assay. Genomic DNA extracted from bulk edited hiPSC by sgRNA1/Cas9 and sgRNA2/Cas9 ribonucleoproteins were amplified by PCR. PCR products were denaturated, re-annealed, treated with (+) or without (-) T7 endonuclease and separated by electrophoresis. Black and grey arrows indicate uncleaved and cleaved DNA PCR products, respectively. The cleavage efficiency indicated below each lane was calculated as the ratio of cleaved amplicons to the total (uncleaved and cleaved) amplicons. **(B)** Sequence alignment of unedited (WT) and edited PCR products obtained from single cell-derived clones. Deletions and insertions are indicated as dashes and green italic letters, respectively. Target sequences of sgRNA 1 and 2 are indicated in blue. PAM sequences are shown in red.

Supplementary Figure S2

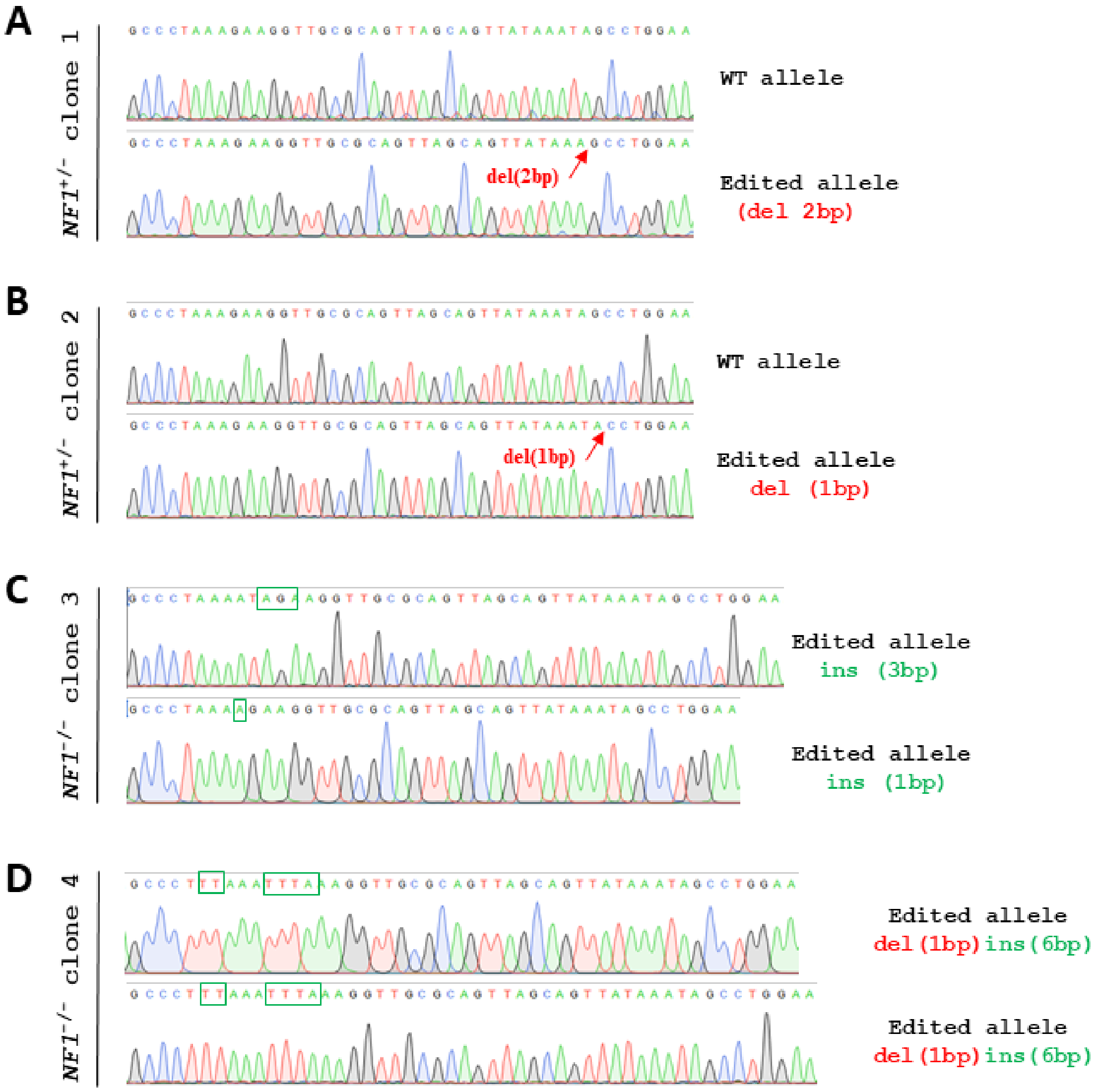

Supplementary Figure S2. Representative Sanger sequencing chromatograms of *NF1* (+/-) and *NF1* (-/-) isogenic hiPSCs. PCR products obtained after amplification of the targeting site of *NF1* (+/-) clone 1 **(A)**, *NF1* (+/-) clone 2 **(B)**, *NF1* (-/-) clone 3 **(C)**, *NF1* (-/-) clone 4 **(D)** isogenic hiPSC were cloned in TA TOPO plasmid and Sanger sequenced. NF1 (+/-) clone 1 or 2 carry a deletion in one single allele at the site targeted by sgRNA2; *NF1* (-/-) clone 3 or 4 isogenic hiPSCs carry an indel mutation in both alleles at the site targeted by sgRNA1. Red arrows: deleted nucleotides, green boxes: inserted nucleotides, ins: insertion, del; deletion.

Supplementary Figure S3

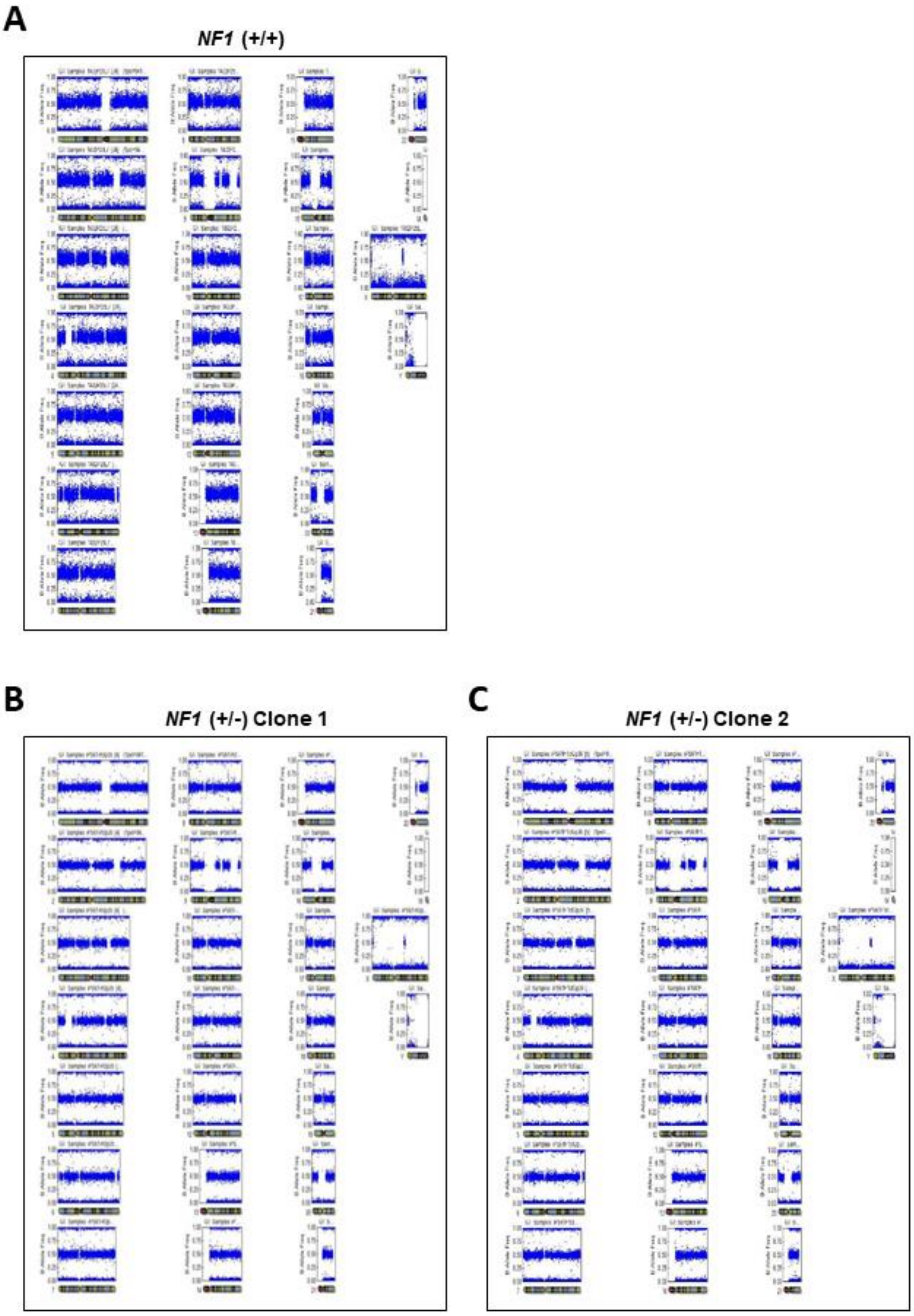

Supplementary Figure S3

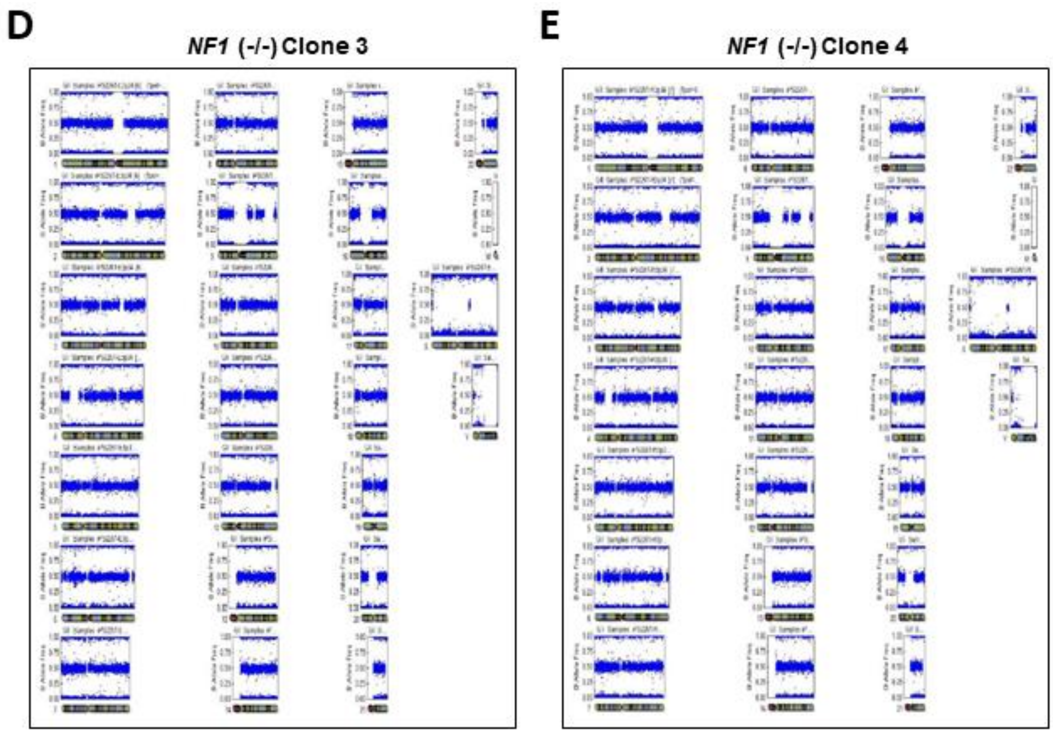

Supplementary Figure S3. Characterization of *NF1* (+/+), *NF1* (+/-) and *NF1* (-/-) isogenic hiPSCs. Genomic SNP array analysis showing *NF1* (+/+) **(A)**, *NF1* (+/-) clone 1 **(B)**, *NF1* (+/-) clone 2 **(C)**, *NF1* (-/-) clone 3 **(D)** and *NF1* (-/-) clone 4 **(E)** hiPSCs

Supplementary Figure S4

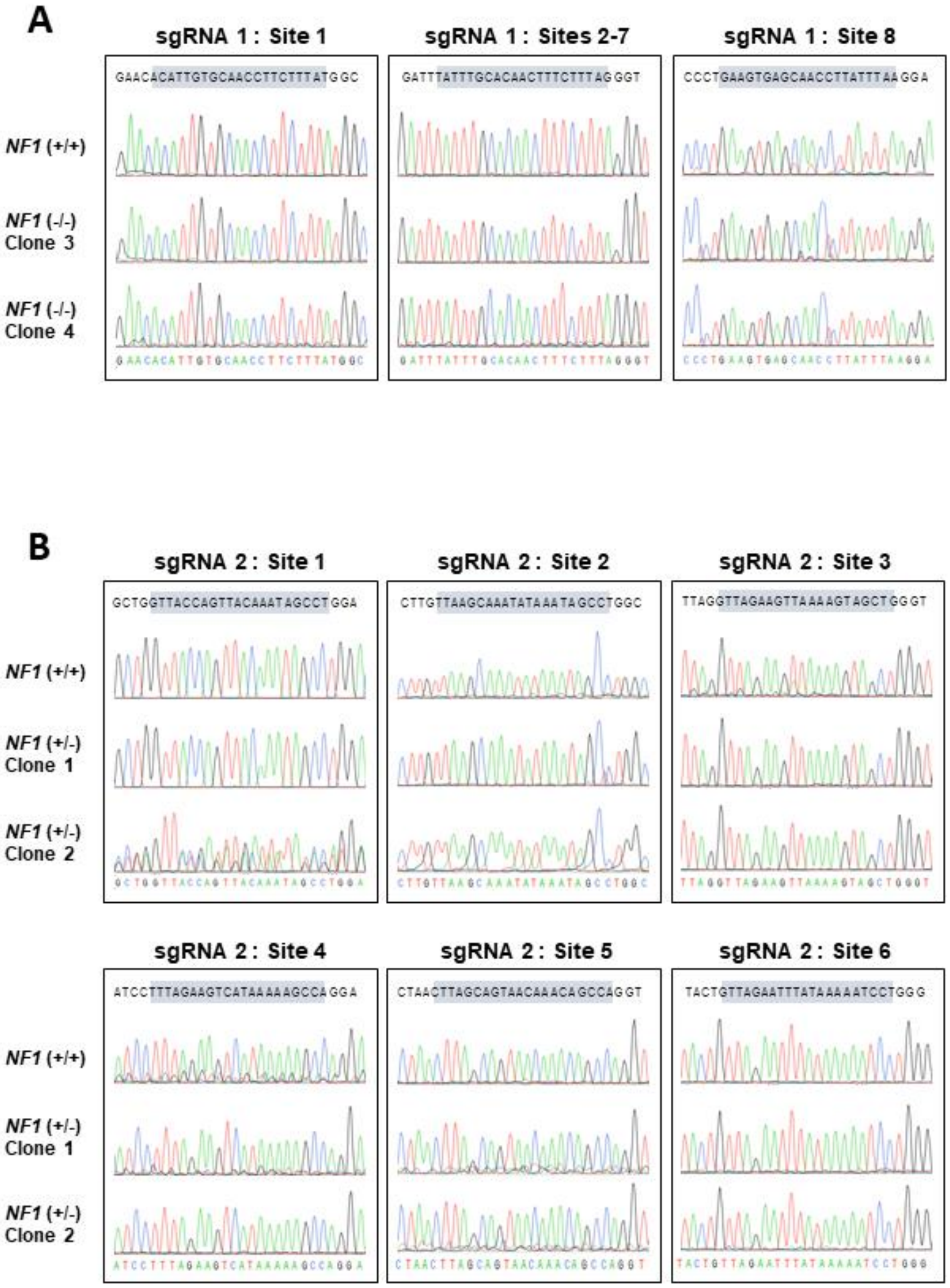

Supplementary Figure S4

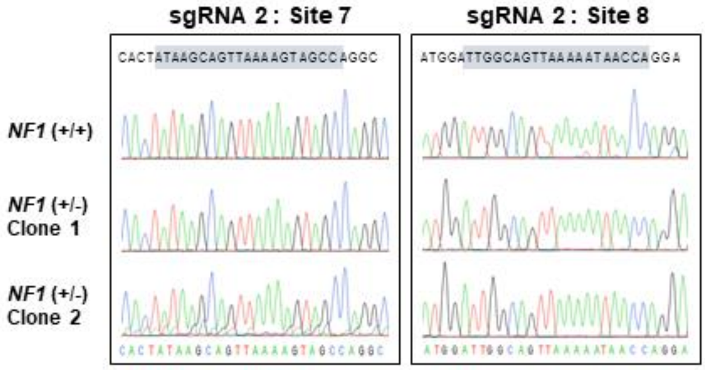

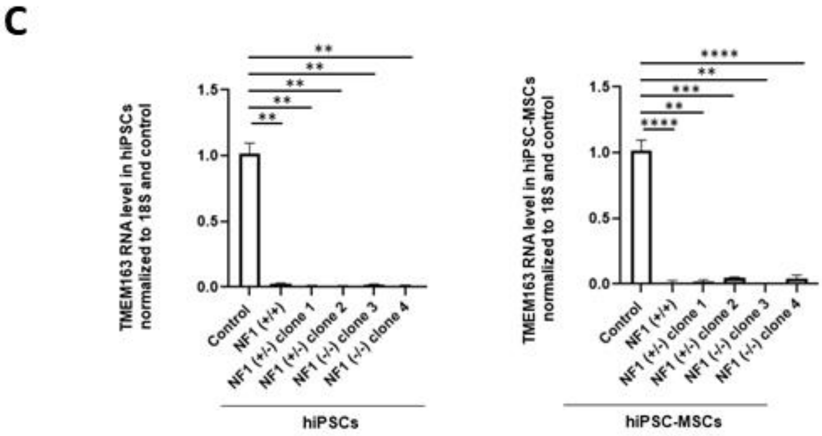

Supplementary Figure S4. Off-target analysis of *NF1* (+/-) and *NF1* (-/-) isogenic hiPSC by Sanger sequencing. Sequence alignment of the top 8 off-target sites identified for the sgRNA1 **(A)** and sgRNA2 **(B)**. *NF1* (+/-) clones 1 and 2 were only edited at the site targeted by sgRNA2; *NF1* (-/-) clones 3 and 4 were edited at the site targeted by sgRNA1. Off-target sites are highlighted in grey. Predicted off-target sites and primers used are listed in Supplementary Table S3 and Supplementary Table S2. For the sgRNA1, the potential off-target sites 2 to 7 correspond to the same sequence but located in 6 different regions of the genome (Supplementary Table S3). The 6 OFT regions were amplified by the same primer set. As the sequences of the NF1 (-/-) clones were equivalent to the sequence of the NF1 (+/+) clone, none of the 6 OFT regions were edited by sgRNA1. **(C)** Analysis of TMEM163 mRNA expression by quantitative RT-PCR in *NF1* (+/+), *NF1* (+/-) and in *NF1* (-/-) isogenic hiPSC (left panel) and hiPSC-MSCs (right panel). Relative mRNA level of TMEM163 is normalized to 18S and expressed relative to the positive control (universal RNA). Statistical analyses are based on a Mann–Whitney nonparametric test for side-by-side comparison. **$p<0.01$, ***$p<0.001$, ****$p<0.0001$,

Supplementary Figure S5

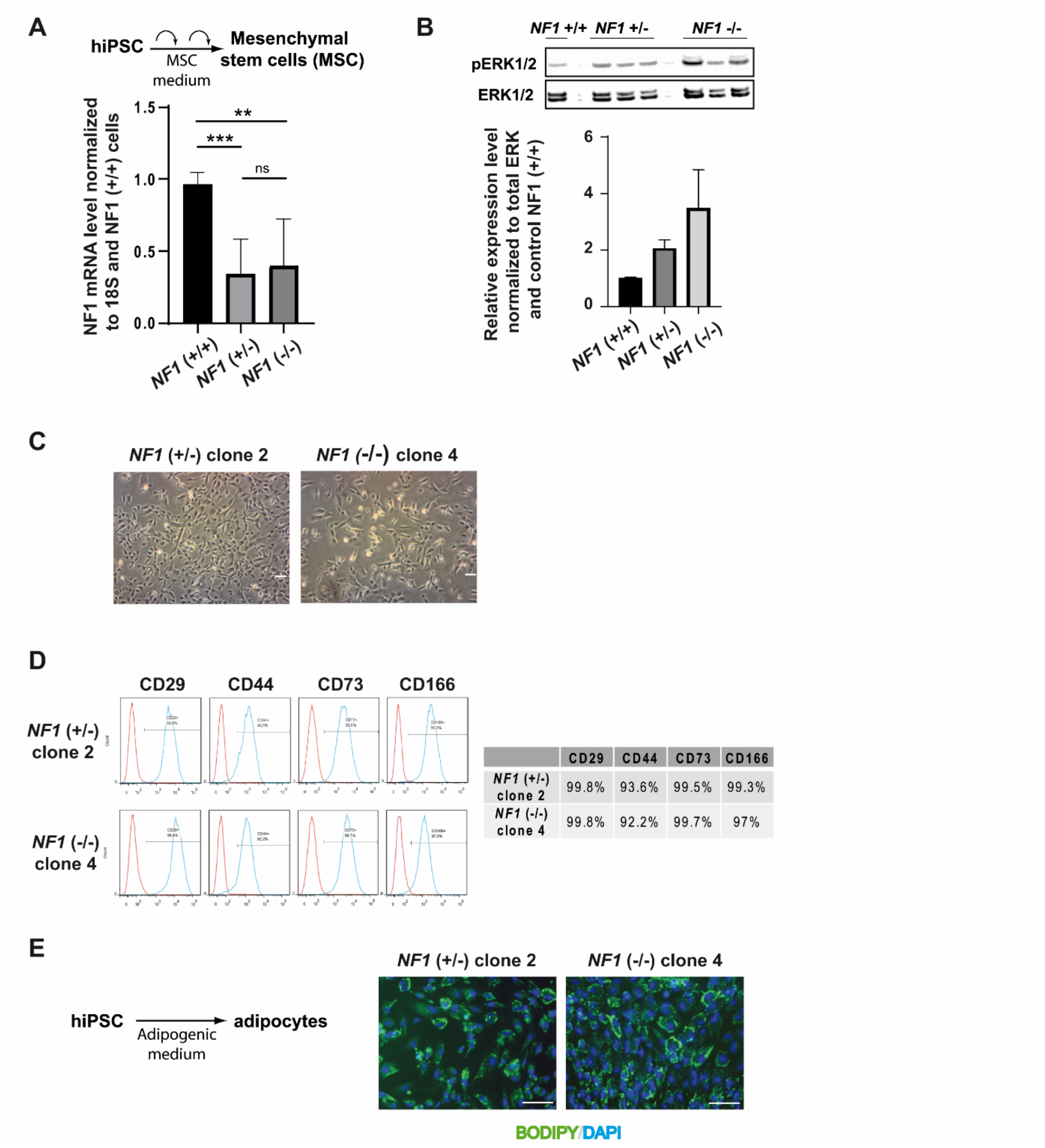

	CD29	CD44	CD73	CD166
NF1 (+/-) clone 2	99.8%	93.6%	99.5%	99.3%
NF1 (-/-) clone 4	99.8%	92.2%	99.7%	97%

Supplementary Figure S5. Characterization of MSC derived from *NF1* (+/-) clone 2 and *NF1* (-/-) clone 2 hiPSCs. (A) Analysis of NF1 mRNA expression by quantitative RT-PCR in *NF1* (+/+), *NF1* (+/-) and *NF1* (-/-) hiPSC-MSCs using primers amplifying exon 26 located in the 3' region of the NF1 mRNA relative to mutations. Relative mRNA level of NF1 is normalized to 18S RNA expression and expressed relative to the *NF1* (+/+) cells. Data obtained from at least 3 independent experiments (2 clones per condition) are represented as mean +/- SD**.** Statistical analyses are based on a Mann–Whitney nonparametric test for side-by-side comparison. **p<0.01, ***p<0.001, ns: not significant. **(B)** Western blot analysis (upper panel) of pERK expression in *NF1* (+/+), *NF1* (+/-) and *NF1* (-/-) isogenic hiPSC-MSCs lines. Quantification of relative pERK levels was normalized by total ERK and *NF1* (+/+) cells (lower panel). **(C)** Phase contrast images of *NF1* (+/-) clone 2 and *NF1* (-/-) clone 4 hiPSCs-MSCs,

Scale bar: 100µm. **(D)** Fluorescence cytometry analysis of CD29, CD44, CD73 and CD166 MSC specific markers in *NF1* (+/-) clone 2 and *NF1* (-/-) clone 4 hiPSC-MSCs. The isotypic staining control peaks are shown in red. **(E)** Images of DAPI nuclear staining (blue) and BODIPY staining (green) performed after 10 days of adipogenic differentiation from *NF1* (+/-) clone 2 and *NF1* (-/-) clone 4 hiPSC-MSCs. Scale bar: 100µm.

Supplementary Figure S6

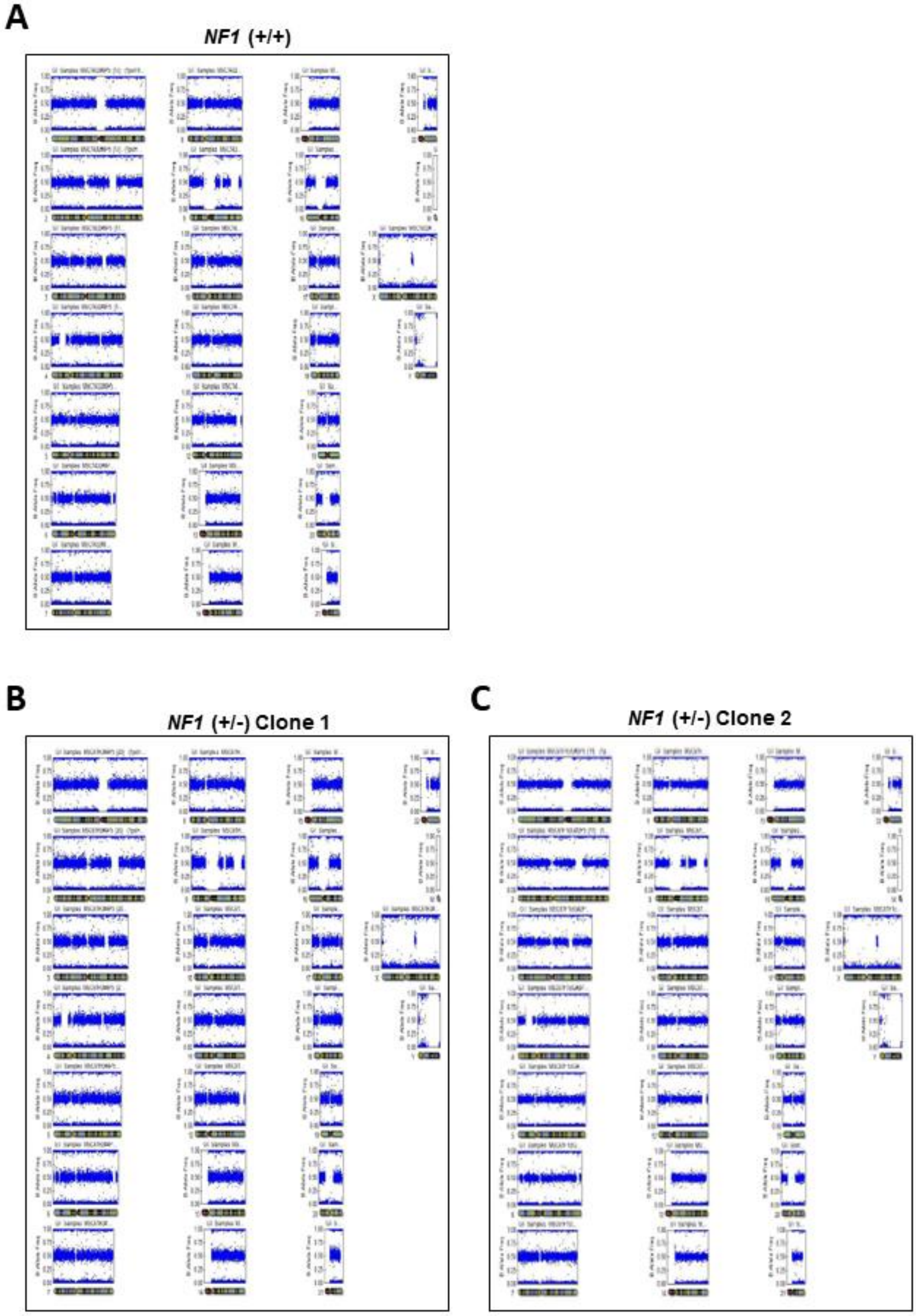

Supplementary Figure S6

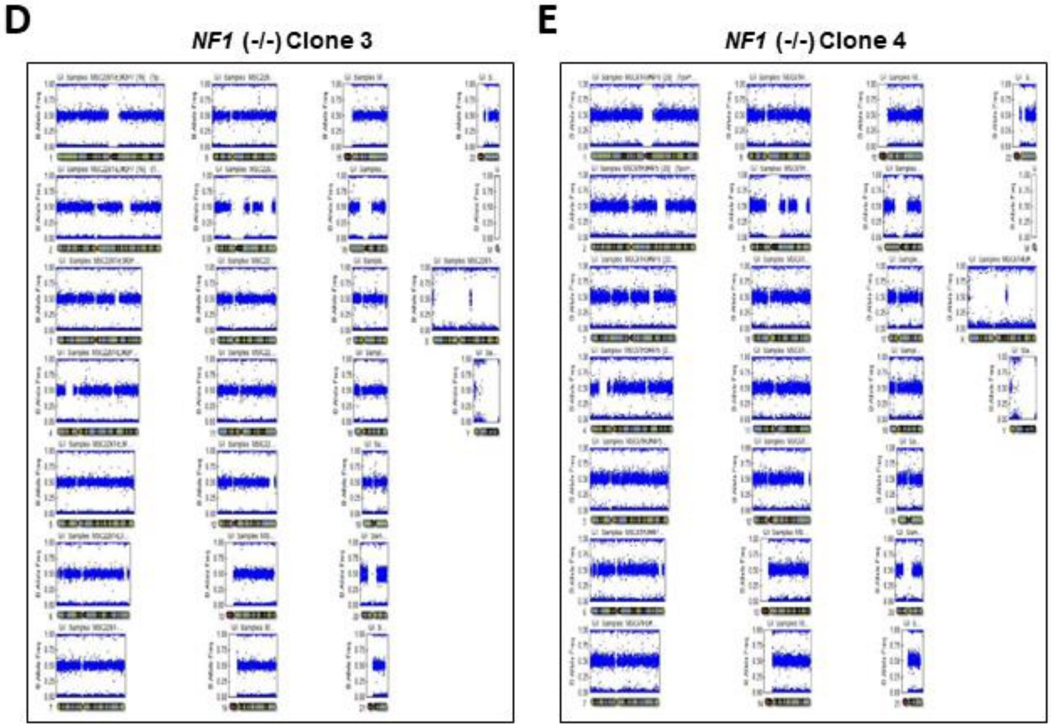

Supplementary Figure S6. Characterization of *NF1* (+/+), *NF1* (+/-) and *NF1* (-/-) isogenic hiPSC-MSCs. Genomic SNP array analysis showing *NF1* (+/+) **(A)**, *NF1* (+/-) clone 1 **(B)**, *NF1* (+/-) clone 2 **(C)**, *NF1* (-/-) clone 3 **(D)** and *NF1* (-/-) clone 4 **(E)** hiPSC-MSCs.

Supplementary Figure S7

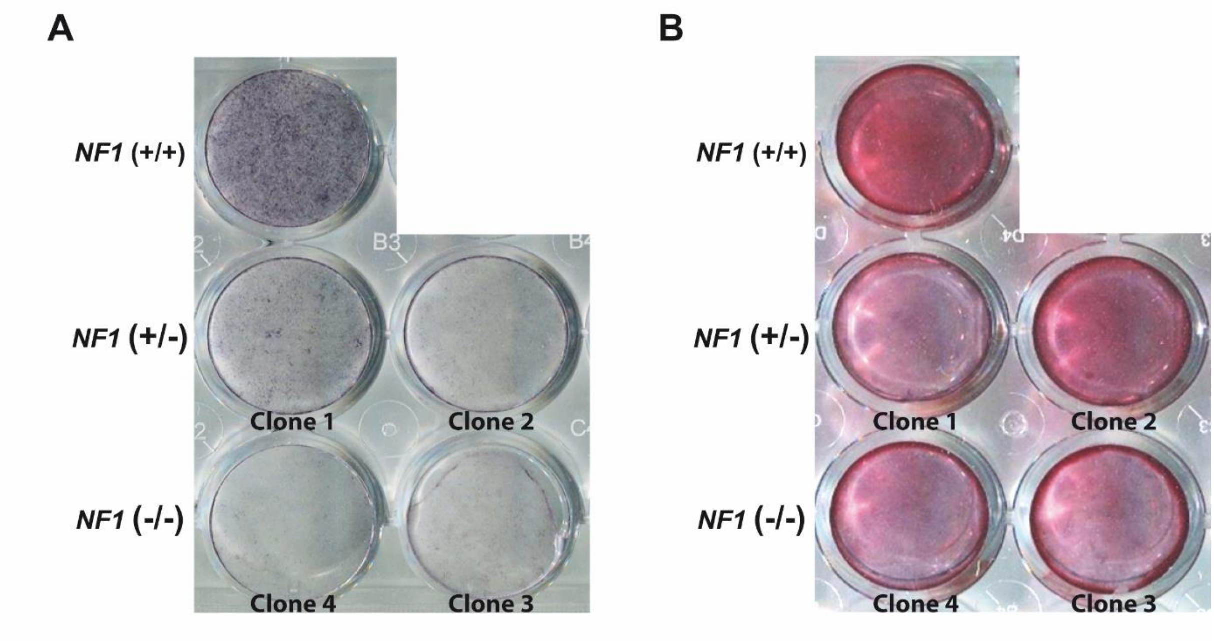

Supplementary Figure S7. Defect in osteogenic differentiation of MSC derived from *NF1* (+/-) clone 1 and *NF1* (-/-) clone 4 hiPSCs.

(A) Macroscopic view of alkaline phosphatase (ALP) staining of *NF1* (+/-) and *NF1* (-/-) hiPSC-MSCs performed after 14 days of culture in osteogenic differentiation medium. **(B)** Macroscopic view of calcium deposition after Alizarin red staining performed 20 days of osteogenic differentiation of *NF1* (+/-) clone 1 and *NF1* (-/-) clone 4 hiPSC-MSCs.

2.2 Supplementary Tables

Supplementary Table S1

List of antibodies used in flow cytometry analysis

Antibody	Supplier	Reference	Dilution
CD29-AF488	Ozyme	#303016	1:20
AF488 Isotype control	Ozyme	#400132	1:20
CD44-APC-H7	BD BioSciences	#560532	1:20
APC-H7 Isotype control	BD BioSciences	#560183	1:20
CD73-APC	Ozyme	#344006	1:40
APC Isotype control	Ozyme	#400119	1:40
CD166-PE	Ozyme	#343904	1:10
PE Isotype control	Ozyme	#400111	1:20
SSEA3-PE	Biolegend	#330312	1:20
PE Isotype control	Biolegend	#400808	1:50
TRA-1-81-AF647	Biolegend	#330706	1:50
AF647 Isotype control	Biolegend	#401618	1:100

Supplementary Table S2

List of primers used for RT-PCR experiments

Gene	Forward primer sequence	Reverse primer sequence
ALP	CAGATGCCAACTTCCCACAC	GGTCCCCTTTCTTGCAGTTG
Col1A	GCTTGGTCCACTTGCTTGAA	ATTTGGGAAGGATGGAGGG
MSX2	TGCAAAACCTATGCTGCCCT	GAACGGGGTATGCTCCAT
NF1	GCACTGTACGGTCCTTGCAA	ATGGAGTGCATGAGACCACTGT
TMEM63	AGAACTACAGGAAGAAGGCATT	ACAGGACAATCGCCGATG

Supplementary Table S3

List of the top 8 potential off-target sites for sgRNA1 and sgRNA2. For the sgRNA1, the potential off-target sites 2 to 7 correspond to the same sequence but located in 6 different regions of the genome. The mismatched bases in putative off-target sites are indicated in red. Off-target sites are sorted by the CFD score.

sgRNA1	Position	Off-target sequence	CFD	Gene	Region
Site 1	chr12_97131705	ACATTGTGCAACCTTCTTTA	0.51		intergenic
Site 2	chr9_41133302	TATTTGCACAACTTTCTTTA	0.39	CBWD7	intron
Site 3	chr9_65732046	TATTTGCACAACTTTCTTTA	0.39	CBWD5	intron
Site 4	chr9_68297996	TATTTGCACAACTTTCTTTA	0.39	CBWD3	intron
Site 5	chr2_113494173	TATTTGCACAACTTTCTTTA	0.39	CBWD2	intron
Site 6	chr9_123014	TATTTGCACAACTTTCTTTA	0.39	CBWD1	intron
Site 7	chr9_65289455	TATTTGCACAACTTTCTTTA	0.39		intergenic
Site 8	chr5_73370984	GAAGTGAGCAACCTTATTTA	0.35		intergenic

sgRNA2	Position	Off-target sequence	CFD	Gene	Region
Site 1	chr2_134568999	GTTACCAGTTACAAATAGCC	0.74	TMEM163	intron
Site 2	chr3_132343064	TTAAGCAAATATAAATAGCC	0.66	ACPP	intron
Site 3	chrX_8990086	GTTAGAAGTTAAAAGTAGCT	0.48		intergenic
Site 4	chr9_26853540	TTTAGAAGTCATAAAAAGCC	0.45	CAAP1	intron
Site 5	chr9_74465822	CTTAGCAGTAACAAACAGCC	0.38		intergenic
Site 6	chr22_29562795	GTTAGAATTTATAAAAATCC	0.35	NIPSNAP1	intron
Site 7	chr4_11386195	ATAAGCAGTTAAAAGTAGCC	0.33		intergenic
Site 8	chr4_11386195	ATTGGCAGTTAAAAATAACC	0.31		intergenic

Supplementary Table S4

List of primers used for off-target analysis for sgRNA1 and sgRNA2.

sgRNA1	Forward primer sequence	Reverse primer sequence
Site 1	GCGACTGCAATGCACGTGGC	GCCTTCCGTTACCTAGTGGCCA
Site 2	CAAAGTCCTTTTCTTGGCTGTT	TGCTTCCTAGAAGAAATACCTTTCA
Site 3	CAAAGTCCTTTTCTTGGCTGTT	TGCTTCCTAGAAGAAATACCTTTCA
Site 4	CAAAGTCCTTTTCTTGGCTGTT	TGCTTCCTAGAAGAAATACCTTTCA
Site 5	CAAAGTCCTTTTCTTGGCTGTT	TGCTTCCTAGAAGAAATACCTTTCA
Site 6	CAAAGTCCTTTTCTTGGCTGTT	TGCTTCCTAGAAGAAATACCTTTCA
Site 7	CAAAGTCCTTTTCTTGGCTGTT	TGCTTCCTAGAAGAAATACCTTTCA
Site 8	CTGGTGATCTGGCAAATGCG	TCGCCTATCTTCCAAGGGGA

sgRNA2	Forward primer sequence	Reverse primer sequence
Site 1	CCATGAGCCCTGTGAGCATT	TAGAGGTTCGATCTGGGCCA
Site 2	CCCCTTGGATTCAGTAGGAG	TAGGATGGATGGAGGCAAAC
Site 3	TGGGAGTGATCACCTGTCTG	CCGGGTGACAGAGTGAGATT
Site 4	GGATGGTAGAAGCAGCAAGG	CCCTTCATCCAGCAAACCTA
Site 5	AGGTTTTGAGAGGCTGACCA	CTCCTAATGCCATCCTTCCA
Site 6	GACAGTAGACCAGGCGCAGT	GCTGGAGTACACTGGCACAA
Site 7	GTTGTGGGGAGGACTCAGAA	TGTTCACTTGGCCACACTCT
Site 8	GCTGCATCAACAAGGCCAAA	GGGGAACAAAAGAAAGGCCC
